# Virtual Online Home-Based Treatment During the COVID-19 Pandemic for Ultra-Orthodox Young Women With Eating Disorders

**DOI:** 10.3389/fpsyt.2021.654589

**Published:** 2021-05-24

**Authors:** Yael Latzer, Esther Herman, Rahel Ashkenazi, Orna Atias, Sofia Laufer, Ateret Biran Ovadia, Tova Oppenheim, Meirv Shimoni, Moria Uziel, Daniel Stein

**Affiliations:** ^1^Faculty of Social Welfare and Health Sciences, University of Haifa, Haifa, Israel; ^2^Psychiatric Division, Eating Disorders Institution, Rambam Health Care Campus, Haifa, Israel; ^3^Eating Disorders Department, Maayanei Hayeshuah Medical Center, Bnei Brak, Israel; ^4^Department of Psychiatry, Sackler Faculty of Medicine, Tel Aviv University, Tel Aviv, Israel

**Keywords:** anorexia nervosa, COVID-19, eating disorders, home hospitalization, online treatment, Jewish ultra-orthodox

## Abstract

**Background:** With the outbreak of the COVID-19 pandemic, the need arose to maintain treatment continuity for religious Jewish Ultra-Orthodox young women with eating disorders (EDs) previously hospitalized in the ED department at the Ultra-Orthodox “Mayanei Hayeshua” medical center in Israel. This need led to the development of home-based online treatment channels, previously unfamiliar, and unaccepted in this population. The implementation of this model had to take into consideration many of the difficulties inherent in the use of online treatment in Jewish Ultra-Orthodox mental health patients.

**Aims:** We sought to investigate our online home-based treatment model implemented during the COVID-19 pandemic in previously hospitalized young Ultra-Orthodox women with EDs.

**Method:** We briefly review the literature on: (1) The Jewish Israeli Ultra-Orthodox culture; (2) Young women in Ultra-Orthodox society; and (3) EDs in Jewish Israeli Ultra-Orthodox women. We then present the inpatient ED department for Ultra-Orthodox young women and describe the online treatment model adapted to this population during the COVID-19 pandemic. We highlight the difficulties, dilemmas, and advantages of our online model with the description of three patients.

**Findings:** Online therapy can serve as a barrier to treatment in some cases, due to physical (lack of suitable online devices except phones), familial (over-crowded families), and religious circumstances, as well as because of the patients' reluctance to take part in this treatment. In other cases, virtual home-based treatment can lead to a positive change. This may be the case in patients who find the distancing online model suitable for them, and in parents who are committed to treatment, using their greater physical and emotional presence at home during the COVID-19 pandemic for the good if their ill-daughters.

**Discussion:** This paper highlights the difficulties and possibilities inherent in a virtual home-based treatment during the COVID-19 pandemic for Ultra-Orthodox young women previously hospitalized because of an ED. This model can be effective for some patients and families if undertaken by a multidisciplinary team that is not only knowledgeable about the treatment of EDs and the use of online strategies but also knowledgeable and culturally sensitive to the specific needs and codes of Ultra-Orthodox populations.

## Introduction

The incidence of eating disorders (EDs) and eating-related pathologies has risen over the past decades, primarily among adolescent girls and young women in modernized Western societies (1). Despite extensive research, the etiology of EDs is still unclear, described in terms of multi-causality, with diverse factors combining to generate and maintain the disorders. Despite the emphasis on genetic, physiological, and neurocognitive factors in the predisposition to an ED, researchers do relate to a culturally-dependent role in the etiology of these disorders (2–4). Moreover, the rate of EDs has also been rising in recent years in less modernized more traditional societies, and in minority groups within Western societies (5, 6).

One of the explanations for these changes in traditional societies is that they are undergoing rapid Westernization due to increasing exposure to Western values. This process is characterized by industrialization, urbanization, globalization, and heightened exposure to Western media in general, and to thin-body ideal messages in particular (7). These sociocultural shifts and the high degree of exposure to Western messages have been linked to growing awareness of weight body-image issues, likely increasing the risk of developing eating-related pathologies and full range clinical EDs (8).

A similar trend of rising rates of EDs in traditional populations has also been observed in Israel (4). Israel is a land of immigrants, home to people from a variety of cultures, religious groups, and ethnicities (9–12). The unique social structure of Israel is characterized by the juxtaposition of ancient traditions with cutting-edge technological development, and of reliance on the dictates of Jewish religion alongside an essentially modern and secular system of legislation. This complex country may offer, thus, an unusual opportunity to study the role of social, religious, and cultural factors amongst continuous stress conditions, in the predisposition to and maintenance of mental disturbances, including EDs.

The treatment of EDs is complex and challenging in view of potentially ambivalent cooperation, often stubborn resistance to treatment, and an inclination to deny the severity of the illness on the part of many young people with EDs (13, 14). The treatment of EDs in young *Haredi* (Ultra-Orthodox Jewish) women is all the more complicated, in that specific culture-dependent difficulties, related to the values and cultural norms of the Ultra-Orthodox Jewish society, are added to the typical denial and lack of cooperation. These include fear of social stigma, worries over impairment of marriage prospects, a preference for resolving problems within the family and community, a tendency to refrain from complaining, and avoidance of treatment in secular institutions to evade the risk of relinquishing the life of Orthodoxy (15). Further issues, related to the treatment itself, may arise from the encounter between modern, Western treatment and therapists and traditional Ultra-Orthodox religious-cultural viewpoints. This disparity may express itself in different models for the perception of sickness and healing, particularly concerning mental health. This tension is heightened by the aspiration of the Ultra-Orthodox community to remain separate from the Israeli secular majority, and by their view of mainstream society and its service providers as incapable of properly understanding and treating their problems (16). These issues may, inherently, pose a significant challenge for treatment (17).

In view of this complexity, a unique inpatient department has been established in 2018 at the Ultra-Orthodox Medical Center “Mayanei Hayeshua,” located in the Ultra-Orthodox city of Bnei-Brak, for the treatment of adolescents and young women from the Haredi sector suffering from EDs. This department is adapted to the specific needs of this population, with the utmost adherence to its values, faith, religious customs, and behavioral codes.

With the outbreak of the covid-19 pandemic in Israel and the resulting imposition of the first of three lockdowns in March 2020, there was an emergent immediate unplanned need to find solutions to maintain the continuity of treatment in this department and preserve the achievements of inpatient care. The first COVID-19-related mandatory lockdown lasted for about 2 months (between 15/03/2020 and 15/05/2020), the second for about 4 weeks (13/09/2020–11/10/2020), and the third for around another 4 weeks (24/12/2020–20/01q2921. In the present study we describe the virtual online treatment in our center during the 1st lockdown. During this period, we treated around 20 patients and families.

Patients and parents rejected the idea of leaving the girls in full hospitalization without the option for family members to visit them or host them on weekends, while the Health Ministry restrictions made a day-hospital format impossible. This necessity led the treatment team of the department to find channels for long-distance online treatment, which has been completely unfamiliar to this population in general, and specifically not accepted in mental health care. In view of the only minimal use of the Internet and any digital means in the ultra-orthodox population, aimed to prevent exposure to Westernized secular messages, the mere acceptance of any online therapy has represented a revolutionary shift. Families have been especially adamant about barring access to the Internet to their children, who are the most easily influenced. This prohibition is so severe that the rule is to turn one's head away when any screen comes into view (18).

Thus, the ED department at the Mayanei Hayeshua Medical Center had to develop a unique innovative virtual model for online therapy adapted to this population. This model had to adhere to the religious values and rules of the society, to be accepted by the religious leaders of the communities (the Rabbis) and by the families.

**The manuscript aimed** to present this specifically developed online therapy model, highlighting the complexities and dilemmas inherent in this treatment, in ultra-orthodox society. The advantages and disadvantages of online therapy within this population are discussed, as a barrier or impetus to therapeutic progress. Three case studies are presented, to illustrate some of the difficulties and advantages of online therapy in young ultra-orthodox women. The concluding discussion summarizes the findings of the long-distance treatment of Ultra-Orthodox young women with EDs during the COVID-19 pandemic and resulting lockdowns. A new culturally sensitive online daycare is proposed for treating young women with EDs of traditional populations in general and Ultra-Orthodox Jewish Israeli communities in particular, during both times of crises such as the COVOID-19 pandemic and at regular times.

## Theoretical Background

### Ultra-Orthodox Jewish Communities in Israel

There is a broad spectrum of religiosity among the Jewish population of Israel, ranging from absolute atheism to the highest devotion to the observance of religious law. The Ultra-orthodox (Haredi) society is a subgroup of the most observant segment along this continuum, with unique cultural, religious, and attitudinal characteristics (19). The ultra-orthodox way of life is reflected in a highly observant approach to religious (rabbinical) authority, a preference to reside in closed Haredi communities, a strict separation of the sexes with specific attitudes to the importance of the family and the highly different roles of men and women, and the limited place of the individual relative to the importance of the community and its values (18).

Jewish Ultra-Orthodox communities maintain their separate educational systems, focused on traditional religious studies, scrupulously avoiding the teaching of general secular core studies, and the exposure to secular media contents including home use of televisions, computers, and the Internet. Ultra-orthodox isolationism is expressed by traditional physical appearance (e.g., beards, sidelocks or head coverings by men, or long sleeves and dresses, as well as wearing wigs by married women), and by adherence to the strictest possible interpretation of the *Halakhah* (religious laws) in front of the secular Israeli law. Every step throughout life is governed by religious edicts and guidance, the purpose of which includes the preserving of a cohesive traditional religious-dominated sociocultural lifestyle. Diligent observation of religious commands and studying in Yeshivas (religious secondary education institutions for men) are spiritual mainstays not to be compromised. Women are expected to support men in achieving these ideals by caring for the household, raising children, and acting as breadwinners for the family. At the same time, women are to be modest—to maintain the principle that their “honor is all inside.” The Ultra-orthodox society is further subdivided into different sects, factions, and “courts” (*hatzerot*), each of which constitutes a separate social structure within the Haredi community at large, with its codes of observance and conduct (17).

### Eating Disorders and Eating-Related Issues in Jewish Ultra-Orthodox Young Women

Research evidence regarding EDs and eating-related issues in religious Jewish subgroups in Israel and elsewhere is meager, particularly concerning the Ultra-Orthodox. Among Jewish Israeli national-religious (“*dati leumi*”) adolescent girls (less religious observance and greater connection to nationalistic Israeli values), higher levels of religiosity have been linked to lower levels of eating pathology (4). Similar findings have also been observed in young American Jewish religious women, in comparison to non-religious young women (20). Another study has found that young women with an internal religious tendency (religious beliefs motivated by inner faith) show lower levels of eating-related pathology in comparison to those with an external religious tendency (religious beliefs motivated by social factors and the wish to belong to the community) (21–23). In a further study, young Jewish American religious women relying more on religious coping patterns in stressful situations have shown reduced eating-related pathology (21).

Similar findings emerged in a recent review of studies that examined body image, attitudes toward eating, eating-related pathology, and dissatisfaction with the body among ultra-Orthodox populations compared to secular and national-religious. A study of body image and body satisfaction using various measurement tools found that ultra-Orthodox women indicated more positive attitudes toward their bodies and expressed less body dissatisfaction compared to secular and modern Orthodox (24). Handelzalts et al. also showed similar findings, in which ultra-Orthodox women had the most positive body image, then modern Orthodox, and at the end of the continuum were secular women (25). Alternatively, another study observed that the highest level of body dissatisfaction was actually among traditional women, compared to other groups, and no differences were found between ultra-Orthodox and secular women (26).

Examining attitudes toward eating and eating-related pathology, no differences were found in attitudes and levels of pathology between ultra-Orthodox, secular, traditional, and Orthodox women (27). As well as in the study of Frenkel et al., using the same tools, no differences were found in the level of eating-related pathology between ultra-Orthodox and national-religious women (28). In a study conducted among adolescent girls aged 14–16 years and from a variety of backgrounds (secular, Christian Arab, and ultra-Orthodox), as well as among control population that included adolescents suffering from anorexia nervosa (AN), the body image was examined using body image Figure drawings (29). Examination indicated similar levels of body dissatisfaction among secular, ultra-Orthodox, and Christian girls, and a lower level compared to the control group of those suffering from AN.

In a study conducted in the United States, attitudes toward eating among ultra-Orthodox and modern Orthodox schoolgirls aged 13–19 years were examined, using the EAT-26 questionnaire. The ultra-Orthodox reported more symptoms of eating disorders, more social pressure (for matchmaking and marriage) compared to modern Orthodox girls. Moreover, the pressure to marry and matchmaking was the main and significant predictor for the onset of eating disorders symptoms (30). In a similar study, also conducted in the United States, EAT-26 examined attitudes toward eating as well as body image, this time among ultra-Orthodox adult women from three streams, modern Orthodox, conservative, and secular, aged 18–70 (31). Unlike the study results among adolescents, no differences were found among adult women in eating-related pathology and symptoms of eating disorders, and these were not found to be related to the level of religious stringency or modesty of dress.

A qualitative study, conducted on six ultra-Orthodox women in South Africa, investigated through semi-structured in-depth interviews thoughts and feelings about the body, as well as the impact of religiosity (32). The study raised five themes about attitudes toward eating, perceptions of body image, peer influence, the influence of the secular outside world, the influence of Judaism, and body image. In general, it was found that the preoccupation with the body and dissatisfaction with its size and features appeared in the same areas and contents as expressed among women from Western culture.

There has been little research among ultra-Orthodox populations in Israel and around the world, especially concerning content related to mental health. This is mainly due to the closure of the ultra-Orthodox communities to the research world, among other things in order not to expose the psychopathology of the population. Therefore, there is great care among the intellectuals who examine the knowledge that is published carefully.

In a recent review (33), all the studies and articles (~180 articles) that were done on eating disorders among ultra-Orthodox society were reviewed, and it was found that most of them were descriptive, with only nine of them indicating quantitative Data. This, to identify culturally related risk factors and protective factors in this population. These examined the literature from 2009 to 2019 and made an in-depth analysis of the nine studies. Risk factors associated with ultra-Orthodox culture included the centrality given to food, low socioeconomic level, strict modesty codes, the importance of thinness for matchmaking and marriage, lack of self-fulfillment, and early marriage, as well as high expectations of a women's role (eshet chayil). The protective factors found included faith, halakhic laws related to awareness and mindful eating, as well as lows that encourages gratitude for food. Moreover, it has been found that covering the body is another protective factor, as part of modesty that reduces the objectification of the body.

These results are consistent with the inclination of Jewish Ultra-Orthodox groups in Israel to use less ED treatment services, relative to their proportional percentage of the population (34). Alternatively, this finding may reflect a lesser inclination of these groups to seek help for mental health-related issues (35). Thus, concealment of psychiatric disturbances is encouraged, aiming to solve the problem within the confines of the community; psychological treatment is to be avoided, especially in mainstream Israeli mental health services because of socio-cultural considerations (36).

Over the past two decades, Ultra-Orthodox young women seem to be experiencing a socio-cultural process of transition and change. As they are increasingly required to support their families financially, they have to seek alternative sources of training and income beyond the traditional teaching and secretarial roles. This has likely led them to greater exposure to mainstream Israeli Westernized messages, including greater exposure to weight-related appearance issues, and the yearning for personal self-actualization and freedom of choice (15). Being in a phase of transition may increase the risk for the development of mental health-related issues, including those related to disordered eating and EDs (37). Indeed, there is seemingly a trend toward rising numbers of adolescent and young Jewish Ultra-Orthodox women hospitalized in recent years in mainstream Israeli specialized ED-treatment departments, likely increasing the fear of their families and communities of greater exposure to secular non-religious influences. This has led to the development, in 2018, of an ED treatment center adapted exclusively to the need of the Ultra-Orthodox population in the “Mayanei-Hayeshuah” Medical Center in the Ultra-Orthodox city of Bnei Brak, Israel.

### Description of the First Department in Israel for the Treatment of Ultra-Orthodox Young Women With EDs

The first Ultra-Orthodox ED department in Israel, located at the Ultra-Orthodox “Mayanei- Hayeshuah” Medical Center in Bnei-Brak, is the realization of the vision of the late director of the Medical Center, Dr. Moshe Rothschild. Realizing the increase in the number of patients with EDs in the Jewish Israeli Ultra-Orthodox population in recent decades, and the problems with secularization and drifting away from the Jewish religious tradition inherent in the hospitalization of young Ultra-Orthodox women with EDs in mainstream Israeli ED treatment departments, Dr. Rothschild decided to set-up in 2018 a specific department for these patients.

This department is specifically designed as a culturally sensitive environment for the treatment of young Ultra-Orthodox women with EDs (as a religious facility, this department does not hospitalize males). The multidisciplinary treatment team includes a child and adolescent psychiatrist (head of the department and the only male in the team), an adult psychiatrist; a pediatrician; psychotherapists (psychologists, social workers, drama, music and movement therapists) nursing staff, clinical nutritionists school staff, occupational therapists, spiritual therapists and support staff for the supervision of eating. Except for the director of the department who is secular non-religious, all other team members are either Jewish Ultra-Orthodox or National Religious. The service includes inpatient, daycare, and ambulatory facilities, treating young women with EDs between the ages of 11–22.

The treatment protocol is based on a behaviorally oriented nutritional rehabilitation program with structured meal supervision Every inpatient receives two weekly individual psychotherapy sessions, a once-weekly family treatment/parental consultation, a once-weekly movement/drama therapy session, and the following group therapies: psychodynamic, cognitive-behavioral (CBT), movement, psychodrama, Jewish religious-spiritual treatment, nutrition, milieu, and parents' group. Adolescent patients have a full school program approved by the Israel Ministry of Education, and young adult patients receive a full rehabilitation program approved by the Israel Ministry of Social Welfare. Treatment for each patient takes into consideration her age and developmental stage.

Because of the Ultra-Orthodox religious orientation of the medical center, the use of smartphones is strictly forbidden, along with any exposure to internet content incompatible with Jewish religious values. Modesty rules apply to both staff and patients, consistent with and respectful of Ultra-Orthodox religious mores. During the course of the treatment, the staff maintains ongoing contact with the religious and spiritual leaders of the families and with the hospital's Rabbis and enlists their help and advice at important junctures. Food is eaten according to strict Ultra-Orthodox Kosher rules. Whereas, most therapies are similar in essence to those in the secular ED department in Israel, the Jewish religious-spiritual treatment group is specifically set up under the premise that enhancement of internal religious orientation (religious beliefs motivated by inner faith) may be associated with a reduction of ED-related pathology (21–23).

### Treatment in the ED Department at the “Maaynei-Hayeshuah” Medical Center During the COVOID-19 Pandemic

The circumstances imposed by the COVID-19 pandemic in Israel with its subsequent mandatory lockdowns required substantial changes in the treatment of patients with EDs. Inpatients could not leave the departments, and visits from the outside were not allowed. Some patients and families preferred to stop inpatient treatment and return home until the end of the lockdown. This was the case in most of the patients treated at the ED inpatient department at the “Maaynei-Hayeshuah” Medical Center. Moreover, daycare and ambulatory services had to be closed, unless in specific conditions. Treatment had, thus, to shift rapidly to telemedicine, being often unfamiliar to Israeli treatment providers in general, not to mention Ultra-Orthodox metal health services. Nonetheless, with the blessing of the Rabbinical authorities, the decision was made to transition the inpatient treatment of patients with EDs to a virtual **home hospitalization** format. This approval was necessary to obtain permission to use an accepted “kosher” Internet system, to enable online therapy.

As noted, exposure to the internet was unfamiliar and often forbidden up to that point, for patients and families, making the provision of treatment with these means highly challenging. At the start, and for some patients throughout the entire COVID-19 period, most of the telecommunication follow-up was conducted with telephone calls; only later, were computer-based video calls and Zoom meetings added.

Treatment with telemedicine in our ED patients encompassed a broad scope of meal and weight measurement supervision, parent counseling, and online individual and group therapy. It was provided by the multidisciplinary staff, working from within the department or from their home. Parents of patients with AN were asked to weigh their daughters once weekly in the local community medical centers during the morning hour. If this was not possible, they were asked to purchase a scale and received guidance from the staff on weighing their daughters weekly, during the morning hours after first urination, with their daughters wearing T-shirt and tights, or a gown.

The virtual online home hospitalization routine consisted of: (1) A weekly online meeting of the entire team staff with the patient and parents, to discuss the achievements and problems of the past week and the goals and challenges for the coming week. (2) Daily monitoring by the department's nurse, to track pharmacotherapy, physiological and emotional condition, everyday functioning, and emerging difficulties. (3) Twice-weekly nutritional counseling, including guidance for patients and parents by the clinical nutritionist for the home-meal supervision. (4) Twice-weekly individual psychotherapy and once-weekly family therapy or parental guidance. (5) A once-weekly psychiatric evaluation. (6) Continuation of group treatment, including the provision of parents' groups. (7) Daily schooling by the educational staff for adolescent patients and continued rehabilitative care for young -adult patients by the occupational therapist and the social worker. The aim of the treatment was to continue with the patients' routine as much as possible, while at the same time to be prepared to manage unexpected crises. The essence of this virtual home hospitalization program enabled such flexibility, tailored to the specific need of each patient and family.

Two psychotherapies deserve a specific consideration. The inclusion of psychodynamic psychotherapy in the treatment regimen is designed to address intrapsychic and interpersonal developmental needs of adolescents often burdened with long-standing illness, in addition to the specific ED-related therapies administered (38). It is of note that other programs in patients with AN have used psychodynamic psychotherapy as their main treatment, showing favorable results (39). Young Ultra-Orthodox girls are usually unfamiliar with the psychotherapeutic language (15). Nonetheless, all psychotherapists were of religious background, thus forming a bridge between psychotherapy and Ultra-Orthodox background. Moreover, dynamic psychotherapy might be specifically required for Young Ultra-Orthodox girls with EDs, who face highly challenging developmental issues, including early settled marriages or a lack of their own self-fulfillment (15).

CBT is provided in this department either as an individual psychotherapy, or in a specifically group format (40), based on Fairburn's “classical” model (41). During the COVID-19 lockdown, each patient read her food monitoring sheets via the online, and other patients and therapists reacted. In this period, group CBT assisted primarily in supporting and expanding the supervised ED-related protocol.

CBT by videoconferencing has been previously found to show good clinical efficacy for ED treatment (42), including in adolescents (43). For example, Waller and associates (44) interviewed 70 clinicians in the field of EDs about their experience with delivery of CBT-ED via telehealth during the COVID-19 period. Some of their tips were akin to our experience. These included the attempt to adhere to our protocol while making the necessary long-distance changes; taking care of privacy considerations as much as possible; discussing the patients' preferences and experimenting with what works best for them; continuing with monitoring; taking care of adequate meals-related supervision and of weighing at home or at the nearest local health services; and providing parental psychoeducational groups, and educating the treatment providers about the proper use of telemedicine.

The families and patients understood the importance of the continuation of treatment and engaged in the process of maintaining prior achievements and preventing regression. To the surprise of the staff, most patients and parents and patients adjusted rapidly to the transition to this hitherto unfamiliar treatment. Overall, most of the patients seemed to adapt to online methods more rapidly than their parents. Some patients responded happily to the invitation and adapted quickly to the change, even surprising the staff by forming a more open connection with their therapists in online sessions, vs. in-person therapy where they tended to remain silent.

Nonetheless, the transition to virtual treatment did not work for everyone, as creating a therapeutic environment at home was sometimes highly challenging. Some patients and families struggled to create this space, particularly when all members of often large families with many children were confined to often relatively small apartments, sharing a single computer, enabling less than optimal privacy. Parents were required to be available to the treatment staff and to clear the path for forming adequate therapeutic environments, maintaining privacy and confidentiality for their daughters, in often overcrowded and highly noisy conditions. Moreover, some patients felt constricted by online therapy and struggled to be open under the difficult conditions in their homes.

Virtual therapy also posted significant challenges for the treatment staff. More than ever, they relied on the parents to create the optimal conditions, under the circumstances, for the supervision of eating, and for the provision of an active presence throughout the day. These experiences and challenges faced by the patients, families, and staff in implementing online home-based therapy gave rise to two main themes: online therapy as a bridge for progress and breakthrough in treatment, and conversely, online therapy as a barrier and detractor from therapeutic achievement.

In the present study we present three case reports about virtual therapy of Ultra-Orthodox patients with EDs and their families during the COVID-19 pandemic and lockdown. Each has reacted differentially to the specific conditions of telemedicine. In this respect, we note that because of the nature of this case-report study, ethics approval was not required by the institution. The demographic and clinical details of the participants have been changed to prevent the identification of patients and families. Verbal and written consent have been obtained for publication from patients and parents.

## Case Study 1

### “The Walls Have Ears”—Home-Based Virtual Therapy Under Impossible Conditions—Virtual Home Hospitalization as a Barrier to Treatment

The following case study illustrates a situation in which online therapy was a barrier to the progress of treatment. S. 13, diagnosed with AN-restricting type is the second of eight children from a Jewish Ultra-Orthodox family. She had a previous brief self-induced restricting eating and weight loss 2 years ago, which was successfully treated on an ambulatory basis. This time, she started complaining of stomachaches, dizziness, weakness, and feeling faint, and thus was taken by her parents to the local community pediatrician. On examination, it emerged that S. had started restricting her eating about a year earlier, losing a significant amount of her weight, unnoticed by those around her. She was immediately referred for pediatric hospitalization in the “Maaynei- Hayeshuah” Medical Center because of bradycardia and low weight. She lost her menstrual period 5 months before her hospitalization. Her weight when hospitalized was 37.250 kg, her height 1.58, and her body mass index (BMI) 15.02 kg/m^2^. After stabilization of her physiological condition, she was referred to our ED department. S. had not been in therapy before. Slowly, she began to form a relationship with her therapist and share her feelings, with a commensurate improvement in her physical and emotional condition. During the COVID-19 crisis, S. was infected with the virus, and sent to isolation at her home, 6 weeks after her admission, where other family members also became ill later. Her weight at that time was 43.150 kg. For 2 weeks the parents put the food near her door, but could not supervise her eating. To maintain the continuity of treatment, the decision was made to transition to online home hospitalization treatment.

The parents, worried that the isolation and cessation of treatment would cause S. to regress, were fully committed to her treatment, allowing her to use their mobile phone devices for that purpose. She was weighed once a week at home, and continued with regular virtual long-distance dialethic nursing, psychiatric and psychological treatment, as well as with her school program. Nonetheless, S. found it difficult to adjust to the digital medium. She struggled especially with feeling safe in front of the video camera in the newly formed environment, being surrounded by her family. She was very tense, had trouble concentrating, and was distracted by the noise around her. S. was particularly concerned with protecting her privacy—“they must not hear,” “they must not listen.” She had to whisper, for fear of being overheard in the next room. She felt that her parents and siblings were eavesdropping on her treatment behind the closed door.

Therapy sessions with S. were continually interrupted. Several times a day, the sessions were cut short because one of her younger brothers entered the room and stood in front of the camera. At other times, her parents came in to have a look despite being asked not to interrupt with the session.

Sometimes, situations of a pause in therapy because of technical problems allowed S. to think of an answer, or, conversely, to be able to avoid answering. S. used the camera in different ways, sometimes making steady, dreamy eye contact, and at other times avoiding eye contact by turning the camera at the ceiling or closet. Difficulties also emerged with her online nutritional management and parental meal supervision, reflected in losing weight, and in the return of maladaptive eating behaviors, already gone when being hospitalized.

During her 6 weeks stay at home because of the COVID-19 lockdown, S. gained only 550 gr. When realizing that online treatment was not improving the condition of S., the decision was made to return her to inpatient treatment, despite the continuation of the COVID-19 pandemic and lockdown. This allowed for better meal supervision and for the individual therapy to be carried out in a setting supporting her privacy and enhancing openness. S. was released from inpatient treatment after around 4 more months. She weighed at that time 48 kg (being in her required weight range), her height increased by 2 cm to 1.60 m, and her menstrual periods resumed after 8 months. It is of note that after her discharge, S. and her family chose to continue with virtual dietetic counseling, but individual psychotherapy continued on a face-to face-basis. In the next months S. slowly regained her weight. In regular conditions she was able to eat independently but felt that she had to had to remind her mother of all her meals. However, when things changed in her routine, for example when her mother had to be near an ill aunt and was not around S. during the day, she quickly and unintentionally “forgot” eating, and lost weight once again.

## Case Study 2

### The Screen as a Safe Encounter: “Far From the Eye, Close to the Heart”—Virtual Online Home-Treatment as an Impetus for Progress and Breakthrough in Therapy

The following case study illustrates a situation in which online therapy may become an impetus for progress and breakthrough in treatment N., 15 years, diagnosed with AN-R, is the fifth of eight children from a Jewish Ultra-Orthodox family. She is an outstanding student, rigorous in her adherence to all religious rules, major and minor alike. As the first daughter after four sons, many of the family's household and caretaking duties were devolved to her, and she undertook these roles devotedly. Parents as well as teachers described her as a good girl, eager to please. Since childhood, N. had been plump, enjoyed eating and was a joyful child. Nonetheless, her weight drew teasing from her brothers.

In the seventh grade, she was weighed by a school nurse, who told her that she was overweight and should seek nutritional treatment. N. decided to lose weight on her own, with extreme restriction of eating. She reduced sugar and fat in her self-induced diet at first, and later also other carbohydrates and proteins. Sensations of emptiness and hunger gave her a feeling of self-control. She subsisted on around 600 calories a day, losing a substantial amount of her weight. Her menstrual cycles stopped at the age of 12.5, after only one period. She was treated in a secular outpatient service, but did not cooperate with her treatment. Eventually, she was hospitalized in an ED department in a mainstream general medical center, because of severe restriction and low weight.

Her weight on admission was 38 kg, her height 1.50 m, and her BMI 16.9 kg/m^2^. N. refused to cooperate with her treatment, and had to be fed with a nasogastric tube. This reluctance was attributed by the department's staff, to a certain extent, to difficulties in the therapeutic encounter because of the gap between the outlook of the nonreligious staff and her Ultra-Orthodox religious mindset. N. was therefore referred to our Ultra-Orthodox department. To avoid the social stigma of full hospitalization, the family requested outpatient care.

On admission to outpatient at age 13.5, she weighed 43 kg; her height was 1.52 m, and her BMI 18.6 kg/m^2^. She was diagnosed with AN-restricting type, with no comorbid psychiatric disorders. She did not gain any weight during ambulatory treatment and had continuous fights with her parents over her eating. Therefore, she was transferred to our daycare service, continuing with her school program. She gained around 2 kgs but was still uncooperative at home and did not eat at school. Consequently, she had to be admitted to inpatient treatment.

At the beginning of inpatient treatment, N. would remain defiantly silent during an entire session, sometimes resting her on the table, yawning, or giving only yes or no answers, rolling her eyes as if to say that she was bored and wished to stop the session. At other times she broke her silence to ask the therapist when the session would be over; still, at other times, she would simply stand up and leave the room, saying that she was tired of the physiotherapist's “digging.” Moreover, N. never entered the room at the scheduled time, claiming that she had forgotten or made other plans. Otherwise, she hid in unexpected places in the department (behind the piano or couch, or under a pile of clothes, blankets, and coats). Time after time, sessions were cut short or canceled altogether. She did not speak with the treatment team during the nutritional counseling, except for short yes/no answers, communicating mainly via her parents.

Seeing that her therapy did not bring to any change, her parents repeatedly asked to change therapists, in the hope of reaching the one who could find her way to N's. heart, but this did not help either. With the other treatment staff, N. communicated only through her parents.

During inpatient treatment N. eventually agreed to receive psychotropic medications for her ED-related anxiety and obsessionally, and was treated with Fluoxetine up to 60 mg/day. Her weight gradually increased to 50,700 kgs being in the range of her target weight, her height increased to 1.56 m. She was still amenorrhoeic, and there was no improvement in her eating at home.

Then, on March 2020, N. tested positive for the COVID-19 and was sent into isolation in her parents' home. Her treatment became online. An initial attempt to conduct her therapy over the telephone was unsuccessful. N. continued her pattern of long silences so that it was unclear whether she was still on the line. This led to the decision to transition to Zoom video calls. To the surprise of the parents and treatment staff, a turning point came at this stage. N. started to speak with the therapist slowly and hesitantly. She looked directly at her therapist for the first time while talking with her. This change was also evident in the different online group therapies, where N, was ready to read aloud her food monitoring sheets. There she expressed her inner struggles with food, weight, and her appearance. The transition to online treatment was accompanied by a significant improvement in N.'s emotional condition. N. She later shared with the team that the physical distance, coupled with the appropriate degree of closeness achieved with the camera, along with the control she gained over her exposure, while close to her family, created a safe, protective environment for her. This enabled N. to become more open to the therapists and her family. Therefore, N. continued with online multimodal treatment even after the release of the mandatory lockdown. In the next months she resumed her menstrual cycles, and was able to maintain her target weight range, but only with the close and active supervision of her parents.

## Case Study 3

### A Family Comes Together to Care for Their Daughter With an ED During the COVID-19 Outbreak:—“*There's no place like home”—*Virtual Online Home Hospitalization as an Impetus for Enhancing Parental Collaboration

The third case study illustrates a situation in which online therapy may become an impetus for recruiting parents to collaborate in treatment. T., 16.5 years old, diagnosed with AN-purging type, with no comorbid psychiatric disorders, is the fifth of eight children from a Jewish Ultra-Orthodox family. T was admitted to inpatient treatment because of severe self-initiated weight loss. She was initially admitted to a pediatric department in a mainstream general medical center, but her family decided to transfer her to our department for religious considerations.

The family reported that the first signs of an ED began around a year ago at the past summer vacation, during the transition from junior high to high school.

T. decided to lose weight, together with some classmates. She cut back on carbohydrates and sugars, gradually reducing her eating to the point of about 500 calories daily. In her words, it was “a regular diet that went out of control.” Eventually, she began to self-induce vomiting after eating. Her parents were helpless, anxious, exhausted, and despairing of the battles surrounding her eating at home, and referred her for inpatient intervention. She had her first menstrual period at about the age of 15. Her period stopped several months before hospitalization.

At admission to our department T. weighed 35.500 kg. Her height was 1.56 m. and her BMI 14.6 kg/${\text{m}}_{..}^{2}$ She denied having any eating or body-image related problems, claiming that she lost weight because of stomach aches and constipation. She gained only around two kgs, and her cooperation with her individual psychotherapy was minimal. Moreover, her parents found it difficult to attend their own treatment because of the burdens of their everyday life.

At the outbreak of the COVID-19 crisis, about a month after T.s hospitalization, the decision was made to begin with online home-based hospitalization. In an earlier attempt to have T. return home for the weekends, her condition deteriorated severely. Nonetheless, both the parents and T. refused the idea of full hospitalization without family visits or being at home during the holy day of “Shabath.” T. received online individual psychotherapy, nutritional, nursing, and psychiatric counseling as well as a full schooling program. The parents received psychoeducation about T.s illness and ongoing guidance and support on meal and post-meal supervision. T.'s parents understood the importance of their meal supervision and were highly committed by being fully present at home, both physically and emotionally. T. was offered the continuation of online individual psychotherapy, but she refused. Being all together at home because of the confinement, with the constant online support and assistance of the department's staff, enabled the parents to supervise T.'s eating behavior closely, making every effort not to be angry, frustrated, and judgmental. Gradually the physiological and emotional condition of T. improved, despite her refusal to receive any individual psychotherapeutic interventions. She was weighed weekly at home in the presence of her mother. When the mandatory lockdown was discontinued, around 6 weeks after its' initiation, T. gained around 4 more kgs, achieving the weight of 43.900 kg. Her period was still not resumed. T and her family decided to continue with online multimodal treatment even after the release of the mandatory lockdown During the next months T. continued to gain weight, and her menstrual period was eventually resumed. She was able to eat relatively independently, but still needed the presence of her mother for reassurance and support.

## Discussion

With the outbreak of the covid-19 crisis and the resulting lockdown, the need emerged to find solutions for the continuation of treatment in adolescents and young women with EDs (45–47). This need led to the adaptation of ED-related treatment to a long-distance online home-based telemedicine format (45–47).

The treatment of EDs, particularly AN is complex, and highly challenging, in view of many physical considerations, often ambivalent cooperation, stubborn resistance to treatment, and an inclination to deny the severity of the illness (13, 14). Treatment may become even more complicated in the case of Jewish Ultra-Orthodox populations, where additional factors related to social values and cultural norms intervene with the treatment. These include fear of social stigma, worries over future marriage prospects of patients and siblings, a preference for resolving problems within the family and close community, and a tendency to refrain from disclosing and complaining. Most importantly, Ultra-Orthodox communities tend to refrain from treatment in Israeli non-religious institutions, to avoid exposure to the norms of mainstream Western secular culture, which might exert a negative influence on young patients and lead them to stray off the path of faith (15).

Further difficulties exist within therapy, in the encounter of Ultra-Orthodox patients and families with modern Western treatment, in that disparities may arise in perceptions regarding the causes for the illness and the ways to treat it (16). These issues pose a significant challenge for treatment in general (15, 17), and for online mental health care in particular. In view of the scant use of the Internet and of digital means in general among the Ultra-Orthodox population, aimed at preventing exposure to secular culture, online therapy, specifically in young women with EDs, represents a revolutionary shift.

The ED department at the “Mayanei-Hayeshuah” Ultra-Orthodox Medical Center in the city Bnei- Brak., Israel attempted to develop a treatment model for home-based virtual online therapy adapted to the specific needs and codes of Ultra-Orthodox populations. This program received the blessing of the parents, the hospital's rabbi, and the spiritual leaders of the families, because of its rigorous adherence to and respect for religious values and rules.

**The objective of this paper** was multifold: to highlight the complexities inherent in virtual online home-based hospitalization for Ultra-Orthodox adolescent and young women with EDS and their families; to describe the dilemmas, disadvantages, and advantages arising in this form of treatment for this population; and to examine whether online treatment did indeed achieve the goals of continuation of treatment and prevention of deterioration.

### EDs During the COVID-19 Pandemic

Current studies indicate an exacerbation of both behavioral and emotional ED-related symptoms with the advent of the COVID-19 pandemic and subsequent lockdowns (46, 48). Patients face in these conditions difficulties related to greater involvement of their families with their eating, and having more unplanned free time, alongside changes in their sleeping routine and screen time, and decreasing outside physical activity (46, 49). These effects may be exacerbated when children are confined to their homes, causing them to have little contact with their peers (49). However, the specific changes in the routine in adolescents with EDs seem to differ from healthy youngsters, showing an increase in physical activity rather than the decrease shown in healthy youngsters, and restricting rather than an increase in the amount of food eaten (49).

Increased eating pathology may be viewed in these circumstances as a means of dealing with a reduced sense of control, in an attempt to regain control over their eating and weight (45, 46). Many patients also report greater anxiety and depression, deterioration in their quality of life, and severe feelings of uncertainty (45, 47, 50). Difficulties related to the necessary changes in a routine because of lockdown and unhelpful social messages may also become triggering (46). In contrast, the absence during the COVID-19 quarantine in youngsters with EDs of intense weight-related comparison driven by social contact, and the reduction in social stressors potentially increasing the social-related anxieties of girls with EDs (51) might reduce the overall distress of some girls.

In Israel, the detrimental effect of the COVID-19 pandemic and subsequent lockdown on the condition of young patients with EDs has been exemplified in the considerably greater number of patients with EDs treated in an ambulatory service in general medical center in Israel [5926 sessions in the first 10 months of 2020, vs. a mean of 4001 sessions in the 5 previous years (52)]. Nevertheless, this increase was accounted for, in part, to the possibility of carrying-out multi-professional telemedicine meetings, comprising of 37% of all sessions during the first 10 months of 2020, vs. no use during the respective period between 2015 and 2019 (52).

Similar to other studies (48, 50), our clinical impression suggests that the COVID-19 resulted in a deterioration of the ED-related condition also in young Ultra-Orthodox girls with EDs. More patients have been hospitalized in our department during 2020 vs. 2019 (35 vs. 25, respectively) and more patient have been hospitalized in the pediatric service in the Maaynei Hayeshuah Medical Center, in 2020 vs. 2019 (12 vs. 7, respectively). Although we have not done any statistical comparison, patients hospitalized in 2020 had ore sever ED-symptomatology, were more suicidal, and showed a greater exacerbation of sexual-trauma related complex post-traumatic stress disorder symptoms. Still it is also our impression that the greater familial support and intervention of Ultra-Orthodox girls with EDs may intervene with the detrimental effects of the COVID-pandemic. We are currently organizing a retrospective study comparing the findings of our ED patients in 2020 vs. 2020.

The impact of the COVID-19 pandemic on the treatment of patients with EDs has been found to vary across treatment facilities and countries. In some ED services, treatment has been either delayed, paused, reduced, or stopped. Therefore, patients have experienced a loss of the required treatment support (45). Other EDs services have been able to continue offering treatment using telehealth and virtual online therapy (49, 52). This has been likely the case also in Israel, where services have provided multidisciplinary long-distance interventions, for outpatient, daycare, and inpatient settings (52).

### Telemedicine for Patients With EDs During the COVID-19

Telemedicine refers to the provision of remote clinical services, via real-time communication, between patients/families and healthcare providers, using electronic audio and visual means. Telemedicine services may expand access by reducing barriers such as travel time, competing responsibilities, or absence from work, and provide advantages for treatment providers and institutions, including schedule flexibility, increased productivity, and less clinic overhead (53).

Indeed, online therapy has been a recognized staple of medical and scientific practice in Western society for years, for mental health care in general and the treatment of EDs in particular, long before the pandemic. Moreover, there is evidence indicating that online treatment has good clinical efficacy for ED therapy (42, 54, 55), including in adolescents (43) that is similar to that of traditional in-person therapy.

With the ongoing COVID-19 pandemic, telemedicine has become useful in decreasing emergency room visits and safeguarding healthcare resources, potentially reducing the spread of the virus (56). In the case of EDs, the role of online treatment has been further augmented with the appearance of the COVID-19 pandemic, in the case of inpatients becoming unable or unwilling to remain in the hospital with the new conditions of the mandatory lockdown (47).

### Telemedicine for Patients With EDs During the COVID-19—The Jewish Ultra-Orthodox Experience

Studies suggest that although online therapy is suitable for many patients, it is of note that these patients are usually from Western cultures (45, 46). In this respect, virtual online treatment is unfamiliar, often unaccepted, and far removed from the realities and religious beliefs of Ultra-Orthodox communities. This may likely lead to unique problems for professionals treating patients in this population, raising difficulties on the level of ethics, Jewish law (“Halakhah”), and religious beliefs. These issues, being already present before the COVID-19 pandemic, when non-religious therapists have treated Ultra-Orthodox patients (15), have been amplified during the COVID-19 era.

Online home-based hospitalization considers that both patients/families and treatment providers are willing to prepare for a new reality, using the tools at their disposal to bring about successful treatment under the present conditions. Thus, patients, families, and therapists come to a shared understanding of the difficulties faced by the patient and agree on the best solutions for adapting therapy to the new conditions. When additional problems arise in connection with value-based prohibitions and restrictions related to the patients' cultural and religious background and beliefs, new unfamiliar treatment methods such as online therapy, with its inherent objective drawbacks, may falter.

Such conflicts are exceptionally complicated in the treatment of Ultra-Orthodox young women in Israel. Their society tends toward isolationism, reflected particularly in the system of rules applied to females. Connection to any form of media, and the Internet, in particular, is either absolutely forbidden, or permitted within severe restrictions to protect against exposure to corrupting “modern” content. The prohibitions on media use are so far-reaching that girls are forbidden to even look at a screen; they are required to turn their heads away if they encounter a screen, so that they are not even tempted to look. This attitude is so deeply ingrained, that any change means a transformation in behavior, beliefs, values, and emotions, potentiality entailing feelings of guilt, anxiety, and powerful resistance from the side of the girl, and especially her family.

Extensive effort is therefore invested in protecting girls who are admitted to inpatient treatment because of their ED from exposure to various forms of media, specifically smartphones, tablets, and any kind of Internet connection. At the same time, the therapeutic setting of our department has the potential to create a safe space for talking privately and revealing oneself in a measured way, without the sense that this constitutes gossip or disrespect of the parents.

After several months of experience with our long-distance treatment model (beginning on March 15th, 2020), two key themes emerged: hospital-based online therapy with Ultra-Orthodox young women might serve for some patients and families as a **barrier to progress in treatment** whereas for other as an **impetus for therapeutic progress and breakthrough**.

**Online treatment as a barrier to therapy**: Difficulties with long-distance treatment may emerge for some patients because of household-related and family-related conditions. Technical issues may arise as most Ultra-Orthodox homes do not have computers or Internet connections; if they do, there is just one computer, generally used by the parents for work. Physical issues may stem from the crowded conditions in most Ultra-Orthodox homes, not allowing intimate conversations and privacy. Family-related issues may involve opposing of online therapy for fear that it might be abused, leading to exposure to content that might lead their ill adolescent daughters, and potentially other children too, to stray to the secular mainstream “path of evil and temptation”. Children, including adolescent ED patients, need their parents' permission to access the internet. The one e-mail address in use belongs to one of the parents and is the only way to send a link for a therapy session, with the parent's approval.

Indeed, the transition to online treatment during the COVID-19 outbreak likely increased the risk of upsetting the fragile therapeutic balance achieved in our department in some patients. For some girls, online treatment provided an outlet for withdrawal and lack of cooperation. Patients blamed their difficulties on online therapy, claiming technical problems with sound, cameras, or internet connections as barriers to the conversation, and intentionally ending sessions before the scheduled time. In other cases, the difficulties emerging seemed genuine associated with household-related difficulties.

These difficulties were partly reflected in case (1). Her patients' parents made a great effort to enable her to receive home-based online treatment. Understanding the importance of continuity of treatment during the COVID-19 lockdown, they were willing to breach the prohibition on the use of the Internet for young girls. Additionally, despite a crowded household, they set up a private corner in one of the rooms and asked the other children to go to another room during therapy sessions. Nonetheless, the struggle with being open and confident in front of the camera in the newly formed environment, unable to use online treatment for her benefit. Her condition deteriorated, eventually requiring her return to inpatient treatment.

By contrast, to the surprise of the multidisciplinary team, some patients with EDs and their families who were highly ambivalent about hospitalization, experienced some relief when offered online home-based hospitalization. Virtual online home hospitalization could be described in such families as an **impetus for recruiting the parents to collaborate in treatment**. Thus, despite the religious restrictions and prohibitions surrounding media use, and the need to care for their ill daughters at home, alongside unfavorable familial conditions, the online treatment option provided at the start a possibility for these parents to refrain from the need to cope with the social stigma and later marriage problems associated with their daughter's hospitalization (36, 57, 58). In this respect, the online program forced by the COVID-19 in our department created a new reality for the family, significantly stimulating the parents' involvement in treatment, actively engaging them in meal supervision, and enabling them to help their daughters directly. Their intensive physical presence at home with their ill daughter, gaining control and flexibility in their availability, alongside the continuous online daily contact with the department's staff, and the immediate possible access to support and guidance, significantly boosted the involvement of the parents in treatment. It is of note that during regular times, the parents were mostly much less at home, unable to provide such a close continuous supervision. These considerations have been highly exemplified in case (3) in our study.

From a different perspective, the inclination of Ultra-Orthodox girls to obey their parents, rooted in the ancient Biblical Ten Commandments, assisted in the empowerment of the parents in the treatment of their ill daughter during the COVID-19 lockdown, rooted in the FBT paradigm (59). This positive change in the condition of young Ultra-Orthodox girls with AN during the lockdown stands in contrast to the deterioration in the ED condition of adolescent girls during the COVID-19 pandemic, even if under treatment, partly related to the greater presence of their parents at home (46). Nonetheless, It is of note that a study performed in Israel in secular female adolescents with EDs during the COVID-19 pandemic, has shown online treatment to be effective in families with positive relationships between the parents and between the parents and their children, but not in families with less favorable familial interrelationships (52). A recent large-scale multicenter study in Italy has also corroborate the contribution of the quality of family relationships on psychopathological changes in patients with EDs related to COVID-19 confinement (50).

In other cases, the online treatment has been found to serve as an **impetus** for progress in the individual psychotherapy of the Ultra-Orthodox adolescent ED patient. In these cases, the virtual treatment created a multifaceted screen in psychotherapy, at times functioning as an escape from exposure, a place to hide, and at other times as a way of controlling the degree of openness. The online screen offered in this respect for the girl a sense of protection and a safe space for the patient, as well las a novel means for an authentic expression.

The healing effects of online psychotherapy were reflected in the second case. N., who grew up with seven siblings, and who had always excelled and pleased everyone, felt, perhaps for the first time, that she had a space just of her own at home, that was safe and protected, allowing her to gain the best of both worlds,—Western modern technology alongside Ultra-Orthodox familiarity and safety. Until that time, she felt driven and managed by others; the only control available for her was to stubbornly resist treatment and entrench herself in the bubble formed by her illness. She “fired” therapists, remained silent during in-person therapy sessions and did everything in her power to bring her treatment to an impasse. The COVID-19 crisis created an unexpected opportunity for change for N., in being able, for the first time, to control the rules and closeness of her psychotherapy.

Nonetheless, one should take into consideration in this case two mitigating circumstances First, the strategic adaptations performed by the family, with the assistance of the treatment staff (60), increased the potential efficacy of online-home-based therapy. Second, there is a trend in recent among young Ultra-Orthodox girls and young women toward greater openness and exposure to modernity, sometimes in secret, including the exposure to various media. The legitimation of online media use because of the COVID-19 conditions, may serve as a release from guilt and shame, providing an opening for self-actualization and self-realization in therapy (15, 61).

Last, the three cases presented here showed different telehealth processes during the COVID-19 period, i.e., barrier to treatment, particularly individual psychotherapy in case (1), impetus to treatment in case (2), and impetus to parental commitment and involvement in case (3). Nonetheless, all shared a specifically high reliance on parental involvement, whether active supervision [case (2) and to a lesser extent case (3)], or passive support [case (1)], with difficulties in being independently responsible for the eating. Thus, the traditional inclination of adolescent Ultra-Orthodox girls, in these cases having an ED, to respect and obey their parents, might interfere with their ability to function independently in specific challenging conditions such as taking care of their ED. Future research in a larger number of patients is required to support this preliminary contention.

Several specific issues have to be considered in telemedicine treatment of young women with EDs. Thus, Rogers et al. (62) have found that video conferencing in these patients may exacerbate body image concerns by increasing their preoccupation with and focusing on their self-appearance. In contrast, there is the issue of the “disembodied environment,” when only the patient's and therapist's faces, but not their bodies, appear on the screen, or when the patient prefers to converse in the session with the camera being closed. What is potentially missing in these cases is body-to-body communication, or the reading of body language (63).

### Practical Considerations and Research Implications

The culturally-sensitive online home-based hospital for young Ultra-Orthodox women with EDs, developed during the COVID-19 pandemic at the “Mayanei Hayeshua” Medical Center, in Bnei-Brak, Israel, represents groundbreaking creative thinking and mutual flexibility on the part of spiritual Rabbinical leaders and the multidisciplinary treatment team of the department to allow for continuity of treatment, preserving progress, and reducing the risk for relapse. Despite the multiple obstacles associated with the use of online treatment in this population, in many cases the program made it possible for the treatment to continue; in some cases, it actually served to stimulate positive changes in treatments previously stalled.

The findings highlight that at least some patients and families found the online intervention acceptable, despite the unfamiliarity of this intervention and the many obstacles described in its implementation. In this respect it is of note that a recent Israeli study (64) found mixed views of patients with EDs treated in a mainstream secular ambulatory service regarding the transition from face to-face to online treatment during the COVID-19 pandemic. This, the majority (68%) of the patients stated that they would not choose to continue online therapy given the option. Longer duration of treatment, stronger therapeutic alliance, and higher COVID-19 anxiety were linked with more positive views toward this transition.

The findings of our study also highlight the need to further develop our model, and to study its long-term effectiveness. In regular times, this format can also be used with Ultra-Orthodox populations finding it difficult and stigmatizing to come openly to a facility, and with families that struggle to cooperate with inpatient treatment because of the hardships and challenges of everyday life. It can also be tailored for young women with EDs who do not feel safe enough in an inpatient setting to disclose themselves; for these women, the virtual screen can serve as a safe privacy-enhancing environment.

We still face many challenges related to the use of online media with Ultra-Orthodox populations, due to their crowded living conditions, and their limited access to virtual communication. In view of the difficulties that have emerged in the online treatment of our patients, the further development of well-protected and easily managed online means is highly recommended. Coordinating this form of treatment with spiritual leaders and obtaining religious decrees is recommended, to avoid later problems and avert worries on the part of patients and their families, that may still consider online treatment as potentially contravening their norms and values.

As noted by several researchers, the use of online services in patients with EDs during and potentially also after the COVID-19 pandemic requires adherence to the guidelines provided by the respective treatment centers in the regular face-to face- management, with the necessary telemedicine-required adaptations (44, 65, 66). Along with their recommendations, we have established a program that takes care both of practical and clinical ethical-related considerations. Nurses daily and pediatricians and psychiatrists weekly, or whenever required, supervise with telehealth facilities (including telephone connections in families not using virtual online services) the medical and psychiatric condition of the patients, and invite them to the hospital whenever necessary. The nurses further assist the parents in the handling of the psychotropic treatment. The nutritionists may assist the parents in weighing and in meal supervision (our model does not advocate direct long-distance supervision of the eating and weighing of the patients by the department's team because of privacy-related considerations that are of particular importance in Ultra-Orthodox populations). We have been able to conduct during the COVID-19 lockdown online CBT groups and parents' psychoeducation groups, as well as individual psychotherapy and team meetings and supervision. All these services could continue, and indeed have continued whenever required, in the post three COVID-19 lockdowns in Israel, altogether lasting for more than a year.

Further quantitative and qualitative research is recommended, to examine the effectiveness of our treatment model over time, and to examine the experiences of patients and their families from their perspective. The importance of understanding the patients'/families' viewpoint vs. that of the treatment providers is linked to differing viewpoints in the perception of illness and healing, particularly concerning mental health. This tension still exists because of the aspiration of the Ultra-Orthodox community to remain differentiated, and by their view of mainstream society and its service providers as incapable of properly understanding and treating their problems (16).

### Limitations

The suggestions of this study should take into consideration its limitations. At the start, it is a descriptive case report study, based on three cases, rather than a structured prospective longitudinal design. Second, the number of patients treated in our department during the COVID-19 period was too small to draw any ststistically-based conclusions about specific ED-related and general psychopathological aspects.

**In conclusion**, our study sought to investigate the complexities and dilemmas inherent in online home-based hospitalization of young Ultra-Orthodox women with EDs. The experience of this unique therapy for this population has demonstrated that online therapy can be a barrier to treatment in some cases, due to physical, familial, and religious circumstances, as well as because of the patients' reluctance to take part in this treatment. In other cases, virtual home-based treatment can lead to positive changes. This may be the case in patients who find the distancing online model suitable for them, and in parents who are committed to treatment, using their greater physical and emotional presence at home for the good of their ill-daughters. For such interventions to be successful, continuous multidisciplinary online supervision and treatment must be carried out by treatment providers who are not only knowledgeable about the treatment of EDs and the use of online strategies, but also knowledgeable and culturally sensitive to the specific needs and codes of Ultra-Orthodox populations.

## Data Availability Statement

The raw data supporting the conclusions of this article will be made available by the authors, without undue reservation.

## Author's Note

With the outbreak of the COVID-19 pandemic, there was a need to maintain treatment continuity for religious Jewish Ultra-Orthodox young women with eating disorders (EDs) that were previously hospitalized in a special ED department for Ultra-Orthodox. This need led to the development of home-based online treatment channels, previously unfamiliar and unaccepted in this population, with difficulties inherent in the use of online treatment.

The present paper aims to present the online home-based treatment model implemented and adapted to young Ultra-Orthodox women with EDs, during the COVID-19 pandemic and to highlight the difficulties, dilemmas, and advantages of this model.

**Our findings showed that** online home-based treatment can serve as a barrier to treatment due to physical (lack of online devices), familial (over-crowded families), and religious circumstances, or as a bridge for change, due to the distancing that this model provides, and the parent's commitment to treatment.

This paper highlights the difficulties and possibilities inherent in a virtual home-based treatment during the COVID-19 pandemic. Additionally, this model can be effective if undertaken by a multidisciplinary team, which is knowledgeable about the treatment of EDs, and the use of online strategies, and culturally sensitive to the specific needs and codes of Ultra-Orthodox populations.

## Author Contributions

YL, EH, and DS contributed to the conception and design and were responsible for the organization of the article. RA, OA, SL, AB, TO, MS, and MU equally contributed to the manuscript. All authors contributed to the article and approved the submitted version.

## Conflict of Interest

The authors declare that the research was conducted in the absence of any commercial or financial relationships that could be construed as a potential conflict of interest.
